# Summer Complaints among Children

**Published:** 1916-07

**Authors:** R. C. Youngblood

**Affiliations:** Falls City, Texas


### Summer Complaints Among Children.

BY R. C. YOUNGBLOOD, M. D., FALLS CITY, TEXAS.

Heat is the causative factor in the majority of cases of summer complaints; first, by devitalizing all the organs of the human body, thereby lessening the power of resistance and interfering with the appetite and digestion; second, heat hastens fermentation and bacteriological changes in the food both in and out of the body. Recognizing these facts, we are in a better position to treat and alleviate the condition.

It is my wish to say a word along the lines of preventive medicine, prophylaxis and sanitation. Most summer complaints are preventive diseases, and it is our good fortune to teach the mothers of the land how prevention can be accomplished. After all, the mother is our friend and co-worker, and without her assistance our results will be practically nil. There is one point upon which we all agree, and that is that the mortality rate is entirely too high.

### MOTHER'S PROPHYLAXIS.

If the mother is not in good physical condition, her health should be restored. If this cannot be obtained, wean the child. Never allow the baby to nurse without first scrupulously cleansing the nipples, nor while the mother's body is in a heated condition. If pregnancy should intervene, wean the child at once. Fatigue, worry, constipation, suppressed menstruation, mental depressions, starvation, improper diet and overindulgence in food or drink; all of these conditions have a deleterious influence on the mother's milk.

### PROPHYLAXIS OF THE CHILD.

During the long drawn out summer and autumn days when the heat is most depressing the "first thought" should be to keep the baby cool and comfortable. This necessitates frequent baths, preferably tub baths, not one but many, through the heated hours of the day. It equalizes the circulation and favors dissipation of heat and moisture, converting an irritable, restless child into a state of quietude and undisturbed sleep.

Teething is a factor in the case by lessening the bowel resistance. Many times the child's resistance is at the breaking point, and the advent of teething terminates in a summer diarrhea. All children have not the same gut resistance, and that accounts for some eating the adult diet without injury, while others seemingly as strong become victims of disease. The pernicious habit some mothers have of feeding their toothless babies all sorts of diet is to be strongly condemned. The child should be clad in a soft, light loosely fitting garment and kept in a cool place whether in or out of doors.

### DIETETIC TREATMENT.

Recognizing that heat plays an important rôle in the ills of infancy and childhood, we must not lose sight of the fact that improper feeding holds a very conspicuous place. The baby's future happiness, health and success depends, in a great measure, upon the parental care and feeding.

The ideal food is the breast milk. But, for various reasons, many mothers are deprived of the proper nourishment for their offspring. These mothers are forced by necessity to accept some form of artificial food. The question that we have asked ourselves countless times is, which one is the best? Wet nursing is out of the question in the country. Mexicans, tenants and poor white people are the principal class that populate the Southwest, and in many cases they are too poor and it is inconvenient to obtain cow's milk, granting it were possible to hand it to them in a prophylactic condition.

Milk is the easiest of all food preparations to become infected; therefore, requiring the utmost care in handling.

Every detail of prophylaxis should be explained to the mother from the time the milk is drawn from the cow until it is placed in the infant's mouth. Or, if it should be artificial food, the same care should be instituted to safeguard the child's health. It is not within the scope of this paper to go into minute details along these lines.

Knowing the environments of the country, I am of the opinion that Borden's Eagle Brand Condensed Milk is the best substitute for the genuine article, of which I have any knowledge. It is safe, clean and cheap, always accessible, easily kept and easily digested. By adding barley water, rice water, oatmeal gruel and occasionally orange juice, or beef juice, this milk provides a well-balanced diet. With this well-balanced diet, we can answer the critic's argument that condensed milk produces rickety children with no lime in their bones, flabby muscles, waxy complexions. Some of the heartiest, most robust children in my town have been reared on this diet.

The artificial food companies have veterinarians who care for the health, food, drink and environments of the cows of their herds. Thus they secure the essentials of good, pure wholesome milk, and by handling it under modern sanitary conditions it is placed on the market fresh, clean and sweet.

The surroundings of the cow in the country are different. The animal, in many cases, has not the green pastures, the running brooks and the protection from the flies; but rather the reverse. Often they are ill fed, badly watered, infested with ticks and annoyed by flies and mosquitoes throughout the entire day. How could we expect the milk to be of good quality? At this writing the mesquite beans of the Southwest are ripe and the stock eat them, rendering the milk unfit for use.

### MEDICINAL TREATMENT.

If there is any tendency in the profession towards therapeutical nihilism among any certain class of diseases, this group without a doubt would hold first place. We run through the category of drugs with the usual results—the easy cases recover; the bad ones die.

For convenience, I have classed summer complaints among children under two diseases—gastroenteritis (or cholera infantum) and ileo-colitis. Ninety-five per cent of summer diarrheas will come under one or the other of these diseases.

### GASTROENTERITIS.

The treatment of these cases depends entirely upon the nature and urgency of the symptoms. In the extreme form continuous bowel movement, of a greenish watery stool, serous matter, and that unmistakable odor, once smelled, never to be forgotten; vomiting at intervals, with eyes sunken, fontanelles depressed, mouth drawn and the skin with a cadaverous appearance are unmistakable signs that these cases demand heroic treatment, positive in character. Nature is working wonders, and in her way is ridding the system of a rank poison. Unfortunately, the body cannot withstand such drastic measures long. The doctor should take the reins in hand and curb nature by giving morphine 1-50 of a grain, strychnine 1-250 of a grain and atropine 1-1000 of a grain hypodermatically, repeating the dose in two hours if necessary. This will check the outpour of water from the system, relieve the sick stomach, lessen the bowel movement and give strength and vigor to the nerve ends. If the body fluids have not been called on too greatly, all will be well and good. But should the little one begin to roll its head from side to side, tossing itself around, as if developing meningeal symptoms, though the general appearance of the child is better, in reality it is preparing to make an exit across the "river Styx."

Should the system gain its equilibrium from the above treatment, it is well to give grain 1-10 to 1-6 of calomel half-hourly for six hours, following with two teaspoonfuls of castor oil. This will secure a thorough emptying of the intestines, and establish hepatic action. After the alimentary tract has been thoroughly cleaned of all excreta and bacteria, and no food has been allowed to enter the body that will favor the propagation of same, further treatment of this disease is very simple.

Zinc sulphocarbolate in 1-6 to 2-grain doses should be given every two hours and continued until convalescence is well under way. And just here a word about this drug: It should be obtained from a reliable chemical company; by so doing the physician will avoid the irritable stomach that is produced by the inferior kind. Zinc sulphocarbolate is given preferably in solution but sometimes it becomes necessary to change the form of administration. Bismuth and pepsin are valuable and will assist in alleviating the irritable stomach. The action of this drug is not thoroughly understood, but why squibble about such matters when the results are ours?

### ILEO-COLITIS.

Gastroenteritis may terminate in an ileo-colitis. Or, the disease may begin with a mild diarrhea, increasing in severity from day to day until it reaches the worst form. Other cases begin by passing mucus and blood. Hence, the confusion in the profession as to making the clinical aspect of the case fit the pathologic findings.

It has been proclaimed from the "house-top" by every writer on summer diarrhea to stop the milk diet at once, whether from the breast or artificial, yet I fear that some fail to heed the warning. The milk diet should not be resumed until the little one is far advanced on the road to Wellville; institute the carbohydrate diet instead. I have reared infants to childhood on such diet. Many children can not take milk at all. But in some instances of bowel disorders of babyhood, it will be found that boiled milk will agree with the child when the raw milk will not.

The same regime of the clean out and keep clean treatment that was applicable to the other forms of summer complaints will apply to this one. The old reliable calomel and castor oil hold first place.

After the treatment is under way, should the temperature advance above 102½° F., it has been may custom to give castor oil, for the fever is invariably caused by retained fecal material or bacteria and its toxin. The oil rids the alimentary canal of the undesirable, leaving the mucosa of the intestine in prime condition.

Where there is mucus, blood and a dark-green stool of frequent evacuations, bismuth subnitrate is of immense value. This should be given in 10 to 20-grain doses every two hours until the disease is well under control and the diet resumed, then less often. Do not expect results with smaller dosage. The inferior quality of this drug will pass from the child in putty-like balls, and instead of being a benefit actual harm will result. The failures attributed to this preparation, in many cases, are due to the impurity of the drug. If black stools do not appear in twenty-four hours after administration, add precipitated sulphur grain 1 to each dose. If bismuth subnitrate is continued for a great length of time, many patients tire of its taking; a change is necessary. If I am giving the powdered form, I shift to the liquid and add the aromatics, or discontinue it for a time. For the tenesmus, paregoric and enemas of normal salt solution and starch water are the sovereign remedies.

Paregoric should be given alone, never in combination with other drugs; given for a specific purpose, and when that has been obtained stop its use. Kerley says: "The worst possible treatment of summer diarrhea is milk in the food and opium in the prescription."

With high fever and with sufficient indication that excrement is being retained in the colon, a colonic irrigation with a normal salt solution will have a happy effect, not only relieving the bowel but reducing the temperature. In the long protracted cases when the vital forces of the system are at a very low ebb, the digestion impaired, and the patient not retaining food or drink, nourishment may be given per rectum with good results. Colonic flushes with astringent solutions have been a failure in my hands, but normal salt and soda have given me excellent results. My mode of administering an enema is with a fountain syringe, hard rubber tip attached, allowing this to pass beyond the internal sphincter muscles. The hips should be elevated four inches; this allows the liquid through gravity to pass slowly into the bowel, presently permeating every fold of the colon back as far as the ileo-cecal valve.

